# Deciphering of seed Health of common food grains (wheat, rice) of North Eastern UP and Gurgaon Haryana, India

**DOI:** 10.1038/s41598-023-34510-3

**Published:** 2023-05-25

**Authors:** Narendra Kumar, S. M. Paul Khurana, Vashist N. Pandey

**Affiliations:** 1 Department of Botany, Guru Ghasidas Vishwavidyalaya (A Central University), Bilaspur, 495009 Chhattisgarh India; 2 Amity Institute of Biotechnology, Amity University Haryana, Manesar, Gurgaon, 122413 Haryana India; 3 Present Address: Experimental Botany and Nutraceutical Lab, Department of Botany, DDU Gorakhpur University, Gorakhpur, 273009 Uttar Pradesh India

**Keywords:** Microbiology, Plant sciences

## Abstract

The stored random samples of food seeds of wheat and rice (60 samples) were purchased from places of Eastern UP and Gurgaon district Haryana. Its moisture contents were estimated. The Mycological investigations of wheat seeds revealed presence of a total number of 16 fungal species viz., *Alternaria alternata*, *Aspergillus candidus*, *Aspergillus flavus*, *A. niger*, *A. ochraceous*, *A. phoenicis*, *A. tamari*, *A. terreus*, *A. sydowi*, *Fusarium moniliforme*, *F. oxysporum F. solani*, *P. glabrum*, *Rhizopus nigricans*, *Trichoderma viride* and *Trichothecium roseum.* While mycological analysis of rice seeds showed presence of 15 fungal species viz., *Alternaria padwickii*, *A. oryzae*, *Curvularia lunata*, *Fusarium moniliforme*, *Aspergillus clavatus*, *A. flavus*, *A. niger*, *Cladosporium* sp., *Nigrospora oryzae*, *Alternaria tenuissima*, *Chaetomium globosum*, *F. solani*, *Microascus cirrosus*, *Helminthosporium oryzae*, *Pyricularia grisea*. It also projected variation in presence of fungal species in blotter and agar plate method of analysis. In wheat Blotter method of analysis showed 16 fungal species while agar plate depicted 13 fungal species. In rice Agar plate method depicted presence of 15 fungal species while blotter method shows presence of 12 fungal species. The insect analysis revealed that wheat samples were infected with *Tribolium castaneum*. While rice seeds sample showed presence of insect *Sitophilus oryzae*. The investigations revealed that *Aspergillus flavus*, *A. niger*, *Sitophilus oryzae* and *Tribolium castaneum* caused reduction in seed weight loss, seed germination, carbohydrate and protein contents of common food grains (wheat, rice). It also revealed that randomly selected A. flavus isolate 1 of wheat showed higher potential of aflatoxin B_1_ production (1392.940 μg/l) while rice isolate 2 showed 1231.117 μg/l production.

## Introduction

Losses of food seeds/grains and other stored products of agriculture due to attack of pests are not a new phenomena. They have been observed by farmers since they became food gatherers from food hunters. So priority should be given to post harvest studies particularly in humid tropical climates. At these regions at least half of the food supply gets lost between harvest and consumption. The deterioration in the stored food commodities occurs because of triple agencies viz. fungi, insects and rodents. Infestation of insects occurs in stored grains and grain products to a variable extent depending upon the storage conditions in developing countries^1^. This is because of lack of appropriate storage facilities and pillaging of grains^2^.

Globally, postharvest losses account for 24% of the total food produced. It varies from about 9% in developed countries to 20% or more in developing countries^3^. According to Wijayaratne et al.^4^, the direct and indirect postharvest losses in humid regions are up to 50%. Losses occurred due to insect infestation in storage are the most serious problem in grain storage, particularly in villages and towns in tropical and subtropical countries, because of humid conditions, poor sanitation and inappropriate storage facilities^5^.

Sometimes molds grow in the insect-infested food grains and these molds produce a chemical substance called aflatoxin which is reported to be associated with the lever cancer of human being^6^.

Overall food grains production was estimated to be 314.51 million tonnes^7^. Rice is staple food for about 800 million people of India. It plays a major role in diet, economy, employment, culture and history. It is the staple food for more than 65% of Indian population contributing approximately 40% to the total food grain production. India grows rice in 43 Mha with production of 112 million tons (Mt) of milled rice and average productivity of 2.6 t −1 ha^8^.

The food seeds (wheat, rice) are stored for varying length of time for various purposes. It is estimated that approximately 10–20% of the stored seeds become deteriorated by fungi. Several fungi have been associated with seeds viz., wheat seed^9,10^, rice seed^11,12^. The non scientific storage of wheat and rice seeds in rural areas of Eastern UP viz., Basti, Deoria, Gorakhpur, Maharajganj and Siddhartha Nagar and Gurgaon district Haryana viz., Farrukhnagar, Manesar, Pataudi, Sohna, Bilaspur leads to heavy deterioration by fungi and insects. However, detailed studies on such deterioration of stored seeds have not been made so far.

Keeping the above in view, in the present investigation, an extensive survey of stored seeds of wheat and rice were made for fungi and insects in order to find out its role in food seeds deterioration. Aflatoxin B1 production of *Aspergillus flavus* isolate were also investigated.

## Materials and methods

### Collection of stored seed samples of food

The random stored samples of food seeds (wheat, rice) [3 to 8 months old ]were purchased from places of Eastern UP viz., Basti, Deoria, Gorakhpur, Maharajganj and Siddhartha Nagar and Gurgaon district Haryana viz., Farrukhnagar, Manesar, Pataudi, Sohna, Bilaspur. From this all 10 selected spots from each spot six samples of food seeds (500 g) were purchased and kept separately in pre-sterilized polyethylene bags after labeling the name of district, tahsil and place. Thus all random 60 samples of seeds purchased were brought to Laboratory for analysis.

### Estimation of Moisture content

Moisture content have role in seed mycoflora. So moisture content were estimated in all randomly collected 60 samples of food seeds (wheat, rice). The weight of 100 seeds (wheat, rice) were recorded randomly using a electronic balance. The seed moisture content was estimated following oven dry method using three replications each of 20 g (ISTA)^13,14^. After estimating the initial moisture content of seeds, about 200 g seed of each sample was kept in muslin cloth bags, to permit free flow of air, and placed in a seed drying room maintaining a constant temperature of 15 °C and 15% RH. Seed samples were drawn at an interval of seven days to estimate the moisture content. The per cent moisture content was estimated.

### Detection of Seed Mycobiota

The mycofloral analysis of all randomly collected 60 samples of food seeds (wheat, rice) were done following techniques (i) Agar plate technique^15^ (ii) Standard Blotter Technique^16^. (i) In agar plate technique, Czapek’s Dox agar medium with following compositions was used during entire experimentation-Ingredients Gms/Litre (Sucrose 30.00; Sodium nitrate 2.00; Dipotassium phosphate 1.00; Magnesium sulphate 0.50; Potassium chloride 0.50; Ferrous sulphate 0.01; Agar 15.00; Final pH (at 25 °C) 7.3 ± 0.2. The medium was sterilized in an autoclave at 20 lb/square inch pressure for 30 min. After cooling of medium to about 40 °C, 10 mg of Streptopenicillin was thoroughly mixed in them in order to prevent the bacterial contaminations^17^. 10 ml of medium was poured aseptically in each of the pre-sterilized petri plates (80 mm diam) separately and allowed to solidify. Glasswares used were pre-sterilized in an oven at 180 °C overnight. (ii) In standard blotter technique, three pieces of blotting paper were sterilized by dipping in ethyl alcohol. These were allowed to dry and placed inside a pre-sterilized Petri plate (80 mm diam).

### Isolation of mycoflora from unsterilized seeds

5 seeds of each sample of food seeds (wheat, rice) were kept equidistantly in each of the pre-sterilized Petri plates containing moistened blotters and solidified agar media separately.10 such assay plates were prepared comprising 50 seeds of each sample. Petri plates were incubated at 28 ± 2 °C for 7 days. The fungi appearing on the seeds were isolated, purified and their single spore cultures were maintained on Czapek’s Dox agar medium in B.O.D. incubator at 10 ± 1 °C.

### Isolation of mycoflora from sterilized seeds

All samples of food seeds (wheat, rice) were surface sterilized by dipping them 0.1%Sodium hypochlorite solution. The seeds were then washed thoroughly with sterilized distilled water to remove the traces of disinfectant. The seeds were placed on moistened blotters and solidified Czapek’s Dox agar medium. The Petri plates were incubated at 28 ± 2 °C. The fungi appearing on the seeds were isolated on the seventh day.

The fungi were identified by comparing their morphological and cultural characteristics with authentic cultures maintained in Mycology Laboratory, Department of Botany, University of Gorakhpur and Amity Institute of Biotechnology, Amity University Haryana as well as with the help of available literature^18–21^.

The per cent frequency of unsterilized and sterilized seeds was calculated by using following formulae- Frequency (%) = No of plates in which individual fungal species occurred × 100/Total no. of plates studied.

### Analysis of food seeds (wheat, rice) collected from different places for insects

Stored random samples (60) of food seeds (wheat, rice) [3–8 months old ] collected from places of Eastern UP viz., Basti, Deoria, Gorakhpur, Maharajganj and Siddhartha Nagar and Gurgaon district Haryana viz., Farrukhnagar, Manesar, Pataudi, Sohna, Bilaspur were also observed for their insect infestation. Observations for the occurrence of insects in samples of stored food grains were recorded as presence (+)/absence (−) of the insect in Table 4.

### Deterioration caused by fungal species

Freshly harvested sterilized wheat, rice seeds were taken in pre-sterilized polyethylene bags (50 g/bag) and these were inoculated by one disc (5 mm diam) of different fungal species separately. For each fungal species 5 control and 5 treatment sets were made and stored for 20 days under laboratory conditions (28 ± 2 °C). The weight loss, germination percentage, Carbohydrate and protein content of treated and control sets were observed. For germination seeds were placed on moistened filter paper and germination was recorded at different intervals for treated and control sets.

The per cent germination was calculated by the following formula:

$$\% {\text{Seed}}\;{\text{germination}} = {\text{No}}{.}\;{\text{of}}\;{\text{seed}}\;{\text{germinated}} \times 100/{\text{Total}}\;{\text{no}}{.}\;{\text{of}}\;{\text{seed}}\;{\text{kept}}\;{\text{for}}\;{\text{germination}}$$

### Deterioration caused by insect species

The living insects viz., *Tribolium castaneum*, *Sitophilus oryzae* were collected in small glass tube (1′ × 4′) and plugged with cotton separately. 50 g surface sterilized healthy seeds of grains (wheat, rice) were placed in sterilized jars (in 5 replicate) with tin covers separately for each commodity.2 pairs of insects (2 males and 2 females) collected in glass tube were introduced in each jar having of one seed such as wheat, rice separately. Sterilized white hard paper strip was placed in jar for each movements of insects separately. A small pin size hole was made in each of the tin covers of the glass jar for gaseous exchange. The jars were placed in dark at room temperature (28 ± 2 °C). The observation in control and treatments were made after 2 months in terms of weight loss, germination percentage, Carbohydrate and protein content in each commodity inoculated seeds separately.

The deterioration caused by fungi/insect in terms of carbohydrate content in wheat and rice seed were studied following Anthrone method^22^. The Carbohydrates were dehydrated through Conc. H2SO4 for forming furfural. Furfural then condenses with anthrone (10-Keto-9, 10 dihydro anthracene) to form a blue-green coloured complex. This was measured through calorimeter at 630 nm. The protein content estimation was done following Lowry et al*.*^23^ by taking bovine serum albumin as standard. The optical density of each chickpea seed sample was taken at 650 nm.

### Detection of aflatoxigenic isolates of *Aspergillus* species

Four isolates of A. flavus were randomly selected from each food seeds samples of wheat, rice separately to determine their Aflatoxin B_1_ producing potential by Thin Layer Chromatography (TLC)^24^. Fifty μl conidial suspension (≈10^6^ conidia/ml) of selected A. flavus isolates were separately inoculated in 49.5 ml SMKY (Sucrose, 200 g; MgSO_4_.7H_2_O, 0.5 g; KNO_3_, 0.3 g; Yeast extract, 7.0 g; Distilled water, 1000 ml) broth medium in 150 ml Erlenmeyer flask and mixed properly followed by incubation at 27 ± 2 °C for 10 days. Content of each flask was filtered after incubation and filtrate was extracted with chloroform (40 ml) in a separating funnel. The separated chloroform extract was dried on water bath at 60–70 °C. The residue left after evaporation was re-dissolved in 1 ml chloroform and 50 μl of it was spotted on TLC plate (20 × 20 cm^2^ of silica gel). The plate was developed in toluene: isoamyl alcohol: methanol (90:32:2; v/v/v) solvent system and intensity of AFB_1_ was observed under ultra violate fluorescence analysis cabinet at an excitation wavelength of 360 nm^25^. The fluorescent blue spots on TLC plate containing AFB_1_ were scraped in 5 ml cold methanol and centrifuged at 3000 rpm for 5 min. Absorbance of supernatant was recorded at 360 nm and AFB_1_ content was quantified^26^.

## Results and discussion

It is evident from Table 1, that the 100 seed weight of wheat and rice seeds were in 4.60 ± 0.23, 2.68 ± 0.13(g) respectively which indicates the seed size diversity. After seven days of incubation grains showed 6.71 ± 0.53, 7.32 ± 0.43, per cent moisture content respectively.

**Table 1:** Weight of seeds of wheat and rice and its moisture content under constant seed drying environment.

Weight of seeds of wheat and rice (100 seeds) [g]		Moisture content in per centage	
		Moisture content on 0 days%	Moisture content on 7th day%
Wheat	4.60 ± 0.23	9.01 ± 0.46	6.71 ± 0.53
Rice	2.68 ± 0.13	9.27 ± 0.33	7.32 ± 0.43

*Values given are mean of three replicates; SD = Standard Deviation.

A total number of 16 fungal species viz., *Alternaria alternata*, *Aspergillus candidus*, *Aspergillus flavus*, *A. niger*, *A. ochraceous*, *A. phoenicis*, *A. tamari*, *A. terreus*, *A. sydowi*, *Fusarium moniliforme*, *F. oxysporum F. solani*, *P. glabrum*, *Rhizopus nigricans*, *Trichoderma viride* and *Trichothecium roseum* were isolated by both agar plate as well as blotter paper methods from 60 random samples of places of Eastern UP viz., Basti, Deoria, Gorakhpur, Maharajganj and Siddhartha Nagar and Gurgaon district Haryana viz., Farrukhnagar, Manesar, Pataudi, Sohna, Bilaspur stored seeds of Wheat (*Triticum aestivum L*.) (Tables 2, 3). In which *Aspergillus flavus*,* A*. *niger*,* A*. *ochraceus* and *A. terreus* were dominant showing 29.0, 27.0, 23.0, 21.0 in blotter and 29.0, 22.0, 22.0, 20.0% in agar plate method of study (Table 4)*.* In wheat seeds the Blotter method of analysis showed 16 fungal species while agar plate depicted 13 fungal species (Table 4). Time to time researchers have isolated fungi the difference may be due to different climatic conditions. 25 genera and 59 species of seed-borne fungi from Egypt with the highest dominance of the genus *Aspergillus* (18 species + 2 varieties), followed by *Penicillium* (12 species + 1 variety, *Fusarium* on third in this regard (5 species + 1 variety), followed by *Rhizopus* spp., *Mucor* spp., *Alternaria* spp., and *Curvularia* spp.^27^; A total of 28 genera and 72 species of seed-borne fungi, most common species viz., *A. niger*, *A. flavus*, *A. terreus*, *A. nidulans*, *A. alternata*, *Cladosporium herbarum*, and *F. oxysporum*^28^; *A. tenuis*^29^; *Chaetomium globosum*, *Drechslera hawaiiensis*, *Fusarium subglutinens* and *Rhizoctonia solani* by using blotter paper method^30^; *Fusarium* spp., *Bipolaris* spp., *Alternaria* spp., *Curvularia* spp., *Aspergillus* spp., and *Penicillium* spp.^31^; *A. flavus*, *A. niger*, *A. alternata,* and *F. verticillioides*^32^*;* A total of 14 genera and 22 species, among which *Drechslera sorokiniana* with maximum mean frequency (18.1%), other pathogenic fungi include *D. tetramera* (15.66), *D. teres* (12.5), *Alternaria alternata* (9.75), *A. tritici* (4.33), *A. triticola* (6.41), *Fusarium semitectum* (10.58), *Cercospora* spp. (2.75) *F. solani* (1.08), *F. oxysporum* (1.66), *Stemphylium solani* (5.66), *S. botryosum* (2.55), *Cladosporium herbarium* (3.41), *Phoma* spp. (6.5) and *Sclerotinia sclerotiorum* (3.25)^33^; *Alternaria alternata* (55.10%), *Bipolaris sorokiniana* (34.69%) and *Cladosporium herbarum* (7.19%)^34^, *Alternaria alternata*, *Aspergillus flavus*, *A. niger*, *Curvularia lunata*, *Fusarium moniliforme*, *Rhizopus stolonifer*, *Mucor* spp. and *Trichoderma viride* from eighty seed samples by using standard blotter paper and agar plate methods^35^; *Alternaria alternata* (infection rate 6.8–19.5%), *Tilletia caries* (1–2%), *Fusarium* spp. (0.5–3.5%), *Cladosporium herbarum* (1.5–3.5%), *Bipolaris sorokiniana* (1.0–4.8%), *Mucor* spp. (1.5–12%), *Penicillium* spp. (0.5– 1.5%), and *Aspergillus* spp. (1–1.5%) on the seeds^36^; Aspergillus, Penicillium, Fusarium, and Alternaria^9^ and Forty-four fungal species belonging to 20 genera, Two prevalent pathogens (average incidence > 40%) Alternaria alternata and Cladosporium spp. Ustilago tritici was present in only seven of the 25 governorates, and less abundant than Tilletia tritici^10^. It is established fact that fungal contamination reduces the viability and ultimately affects the germination of the wheat seeds^37^.

**Table 2:** Number of Fungal species recorded from Wheat (*Triticum aestivum *L.) and Rice (*Oryza sativa* L.).

Fungal Species Recorded	Wheat (*Triticum aestivum *L.)	Rice (*Oryza sativa *L.)
*Alternaria alternata* (Fr.) Keissler	+	−
*Alternaria padwickii* (Ganguly) M.B. Ellis,	−	+
*Alternaria tenuissima* Samuel Paul Wiltshire	−	+
*Aspergillus candidus* Pers ex	+	−
*Aspergillus clavatus* Desm	−	+
*Aspergillus flavus* Link	+	+
*Aspergillus niger* van Tieghem	+	+
*Aspergillus ochraceous* Wilhelm	+	−
*Aspergillus oryzae* E. Cohn	−	+
*Aspergillus sydowi* (Bainier and Sartory) Thom and Church	+	−
*Aspergillus tamarii* Kita	+	−
*Aspergillus terreus* Thom	+	−
*Chaetomium globosum* Kunze	−	+
*Cladosporium herbarum* (Pers.) Link	−	+
*Curvularia lunata* (Wakker) Boedijn	−	+
*Fusarium moniliforme* Sheldon	+	+
*Fusarium oxysporum* von Schlechtendal	+	−
*Fusarium solani* (Mart.) Sacc	+	+
*Helminthosporium oryzae* Breda de Haan	−	+
*Microascus cirrosus* Zukal	−	+
*Nigrospora oryzae* (Berk. & Broome) Petch	−	+
*Penicillium glabrum* (Wehmer) Westling	+	−
*Pyricularia grisea* Sacc	−	+
*Rhizopus nigricans* Ehr	+	−
*Syncephalastrum racemosum* Cohn	+	−
*Trichoderma viride* Pers.ex.Fr	+	−
*Trichothecium roseum* (Persoon)	+	−
Total	16	15

**Table 3:** Number of Fungal species recorded from different places of Eastern Uttar Pradesh and Haryana.

Fungal species recorded	Basti, U.P	Deoria, U.P	Gorakhpur, U.P	Maharajganj, U.P	Siddhartha Nagar, U.P	Gurgaon, U.P	Farrukhnagar, Haryana	Manesar, Haryana	Pataudi, Haryana	Sohna, Haryana	Bilaspur, Haryana
*Alternaria alternata* (Fr.) Keissler	−	+	+	+	−	+	+	+	−	+	−
*Alternaria padwickii* (Ganguly) M.B. Ellis,	+	+	+	−	+	+	−	+	+	−	+
*Alternaria tenuissima* Samuel Paul Wiltshire	+	−	−	+	+	+	+	+	+	−	+
*Aspergillus candidus* Pers ex	+	+	+	+	−	+	+	+	+	+	−
*Aspergillus clavatus* Desm	−	+	+	+	+	−	+	−	+	+	−
*Aspergillus flavus* Link	−	+	+	−	+	+	+	−	−	+	+
*Aspergillus niger* van Tieghem	−	+	+	−	+	+	−	+	+	+	−
*Aspergillus ochraceous* Wilhelm	+	−	−	+	+	+	+	−	−	+	+
*Aspergillus oryzae* E. Cohn	−	+	+	−	−	+	+	−	+	−	+
*Aspergillus sydowi* (Bainier and Sartory) Thom and Church	+	−	+	+	−	+	+	+	+	−	+
*Aspergillus tamarii* Kita	+	−	+	+	−	+	−	+	+	+	−
*Aspergillus terreus* Thom	−	+	+	+	+	−	+	+	−	+	+
*Chaetomium globosum* Kunze	+	−	+	+	+	+	−	+	+	−	+
*Cladosporium herbarum* (Pers.) Link	+	+	−	−	+	+	+	−	+	+	−
*Curvularia lunata* (Wakker) Boedijn	+	−	+	+	+	−	+	+	+	−	+
*Fusarium moniliforme* Sheldon	−	+	+	−	+	+	−	+	+	−	+
*Fusarium oxysporum* von Schlechtendal	+	+	−	+	+	−	+	−	+	−	+
*Fusarium solani* (Mart.) Sacc	+	−	+	−	+	+	−	+	−	+	−
*Helminthosporium oryzae* Breda de Haan	−	+	+	+	−	+	+	−	+	+	−
*Microascus cirrosus* Zukal	+	−	+	−	+	+	+	+	+	−	+
*Nigrospora oryzae* (Berk. & Broome) Petch	−	+	+	+	+	+	−	+	+	+	−
*Penicillium glabrum* (Wehmer) Westling	+	+	−	+	+	+	+	+	+	−	+
*Pyricularia grisea* Sacc	+	+	−	+	+	−	−	+	+	+	−
*Rhizopus nigricans* Ehr	+	+	+	−	−	+	+	+	−	+	+
*Syncephalastrum racemosum* Cohn	−	+	+	+	+	−	−	+	+	+	+
*Trichoderma viride* Pers.ex.Fr	+	−	+	+	+	−	+	+	+	+	−
*Trichothecium roseum* (Persoon)	+	+	−	+	+	−	−	+	+	+	+
Total	17	18	20	18	20	19	17	20	21	17	16

+: Presence of fungal species; −: Absence of fungal species.

**Table 4:** Percent frequency of isolated mycobiota from of stored seeds of Wheat (*Triticum aestivum* L.) from places of Eastern UP viz., Basti, Deoria, Gorakhpur, Maharajganj and Siddhartha Nagar and Gurgaon district Haryana viz., Farrukhnagar, Manesar, Pataudi, Sohna, Bilaspur.

Fungi recorded	Unsterilized seed		P-value	Sterilized seed		P-value
	Blotter method	Agar plate method		Blotter method	Agar plate method	
*Alternaria alternata* (Fr.) Keissler	3.1 ± 0.16	5.1 ± 0.08	0.000059	2.1 ± 0.10	–	0.0000097
*Aspergillus candidus* Pers ex	2.1 ± 0.04	4.1 ± 0.09	0.0000099	–	–	–
*A. flavus* Link	29.0 ± 0.13	29.0 ± 0.14	1.0	4.6 ± 0.15	9.7 ± 0.11	0.00000012
*A. niger* van Tieghem	27.0 ± 0.12	22.0 ± 0.15	**0.000997**	3.7 ± 0.13	7.7 ± 0.19	0.0000004
*A. ochraceous* Wilhelm	23.0 ± 0.10	22.0 ± 0.07	**0.159902**	4.6 ± 0.13	7.7 ± 0.09	0.0000028
*A. tamarii* Kita	3.0 ± 0.12	3.2 ± 0.17	**0.070484**	–	–	–
*A. terreus* Thom	21.0 ± 0.45	30.6 ± 0.33	**0.000225**	4.5 ± 0.40	6.7 ± 0.11	0.000069
*A. sydowi* (Bainier and Sartory) Thom and Church	5.3 ± 0.16	5.0 ± 0.18	0.06532	2.1 ± 0.19	1.0 ± 0.18	**0.001041**
*Fusarium moniliforme* Sheldon	3.1 ± 0.11	5.1 ± 0.17	0.0000064	2.0 ± 0.13	–	0.00000414
*F. oxysporum* von Schlechtendal	3.1 ± 0.21	3.1 ± 0.37	1	2.1 ± 0.43	1.4 ± 0.44	**0.000193**
*F. solani* (Mart.) Sacc	3.1 ± 0.05	3.0 ± 0.44	**0.327928**	2.1 ± 0.26	1.2 ± 0.29	**0.000571**
*Penicillium glabrum* (Wehmer) Westling	5.1 ± 0.22	2.3 ± 0.24	0.0000048	–	–	–
*Rhizopus nigricans* Ehr	3.1 ± 0.29	0.3 ± 0.28	0.0000003	–	–	–
*Syncephalastrum racemosum* Cohn	4.1 ± 0.41	–	0.0000006	–	–	
*Trichoderma viride* Pers.ex.Fr	3.0 ± 0.35	–	0.0000001	–	–	–
*Trichothecium roseum* (Persoon)	3.4 ± 0.25	–	0.000000004	–	–	–

–: Absence of fungal species; US: Unsterilized seeds; SS: Sterilized seeds.

*Values given are mean of three replicates; SD = Standard Deviation.

Significant values are in bold.

The fungal investigations on 60 samples of different stored food seeds of rice (*Oryza sativa* L.) from places of Eastern UP viz., Basti, Deoria, Gorakhpur, Maharajganj and Siddhartha Nagar and Gurgaon district Haryana viz., Farrukhnagar, Manesar, Pataudi, Sohna, Bilaspur showed presence of 15 fungal species viz., *Alternaria padwickii*, *A. oryzae*, *Curvularia lunata*, *Fusarium moniliforme*, *Aspergillus clavatus*, *A. flavus*, *A. niger*, *Cladosporium* sp., *Nigrospora oryzae*, *Alternaria tenuissima*, *Chaetomium globosum*, *F. solani*, *Microascus cirrosus*, *Helminthosporium oryzae*, *Pyricularia grisea*. Out of these fungal species *Aspergillus flavus*, *A. niger* was found to be dominant on the basis of per cent frequency. Agar plate method depicted presence of 15 fungal species while blotter method shows presence of 12 fungal species. Agar plate method showed higher per cent frequency while blotter method showed lower frequency of fungal species (Table 5). Time to time researchers have isolated fungi the difference may be due to different climatic conditions viz., *Curvularia*^38^; *Alternaria alternata*, *A. tenuissima*, *Aspergillus niger*, *A. flavus*, *A. terreus*, *Chaetomium globosum* and *Curvularia lunata*^39^; *Drechslera oryzae*, *Alternaria padwickii.*^40^; *Gibberella zeae* (anamorph, *Fusarium graminearum*) and *Fusarium semitectum*, *with F. acuminatum*, *F. anguioides*, *F. avenaceum*, *F. chlamydosporum*, *F. equiseti*, and *F.* oxysporum^41^; *A. padwickii*, *A. longissima*, *Curvularia oryzae*, *C. lunata*, *Drechslera oryzae*, *A. niger*, *Fusarium moniliforme*, *F. semitectum*, *F. oxysporum*, *F. solani* and species of *Phoma*, *Cercospora*, *Chaetomium*, *Sclerotium*, *Penicillium*, *Myrothecium* and *Colletotrichum* from seeds of rice varieties^42^; *Bipolaris oryzae*, *Fusarium moniliforme*, *Pyricularia oryzae*, *Rhizoctonia solani*, *Sarocladium oryzae*, *Sclerotium oryzae*, *Microdochium oryzae*, *Curvularia lunata* are associated with seed infection of rice causes yield reduction, quality deterioration and germination failure^43^; Totally 8 genera of fungi viz., *Alternaria*, *Aspergillus*, *Bipolaris*, *Chaetomium*, *Curvularia*, *Fusarium*, *Sarocladium* and *Trichoderma* comprising twelve species^44^; *Helminthosporium oryzae*^45^; *Penicillium globosum*, *Rhizoctonia* sp., *Phoma* sp. were isolated in higher frequency from blotter paper method and *Curvularia lunata* and *Drechslera* sp. from agar plates^46^; *Alternaria padwickii*, followed *by Curvularia lunata*, (5.9–14%) *Fusarium oxysporium* (9.9–13.5%) and *Verticillium* sp (2–9.5%)^47^; six genera viz. *Bipolaris oryzae* (2.5 to 8.53%), *Alternaria padwickii* (5.3 to 13.35%), *Fusarium moniliforme* (11.66 to 21.67%), *Fusarium oxysporum* (1.25 to 4.35%), *Curvularia lunata* (1.95 to 7.5%) and *Aspergillus* sp.(1.75 to 6.54%)^48^; *Alternaria padwickii*, *Curvularia lunata*, *Fusarium moniliforme*, *Helminthosporium oryzae*, *Sarocladium oryzae*, *Pyricularia oryzae*, *Rhizopus oryzae*, *A. niger* and *Trichoderma* sp.^49^; *Penicillium* sp. and *Aspergillus* sp.^50^; *Aspergillus flavus*, *A. niger*, *Penicillium* sp. and *Fusarium* sp.^11^; *Aspergillus* sp., *Fusarium* sp., *Rhizopus* sp., *Gibberella* sp., *Tilletia* sp. and *Penicillium* sp.^12^. As evident from Table 4, the wheat samples collected from Basti, Deoria and Maharajganj, Farrukhnagar, Manesar, Pataudi consisted of *Tribolium castaneum*. Sohna and Bilaspur rice sample showed absence of insect *Sitophilus oryzae*. It is interesting to note that sample of food seeds of Basti was badly infested from where maximum insect population was recorded (Table 6).

**Table 5:** Percent frequency of isolated mycobiota from of stored seeds of rice (*Oryza sativa* L.) from places of Eastern UP viz., Basti, Deoria, Gorakhpur, Maharajganj and Siddhartha Nagar and Gurgaon district Haryana viz., Farrukhnagar, Manesar, Pataudi, Sohna, Bilaspur.

Fungi recorded	Unsterilized seed		P-value	Sterilized seed		P-value
	Blotter method	Agar plate method		Blotter method	Agar plate method	
*Alternaria padwickii* (Ganguly) M.B. Ellis,	1.0 ± 0.16	3.1 ± 0.24	0.000018	1.1 ± 0.14	–	**0.000126**
*Alternaria tenuissima* Samuel Paul Wiltshire	–	2.2 ± 0.06	0.000000045	–	–	–
*Aspergillus clavatus* Desm	1.0 ± 0.09	1.1 ± 0.08	**0.481817**	–	–	–
*A. flavus* Link	27.0 ± 0.24	37.1 ± 0.17	0.00000057	13.9 ± 0.22	9.3 ± 0.16	0.00000048
*A. niger* van Tieghem	24.0 ± 0.22	29.1 ± 0.23	0.00000039	14.9 ± 0.24	9.1 ± 0.24	0.00000012
*A. oryzae* E. Cohn	5.2 ± 0.16	11.1 ± 0.24	0.000000053	4.7 ± 0.14	4.7 ± 0.28	1
*Chaetomium globosum* Kunze	–	2.3 ± 0.17	0.000000019	–	–	–
*Cladosporium herbarum* (Pers.) Link	–	9.9 ± 0.14	0.00000011	–	6.1 ± 0.16	0.000000001
*Curvularia lunata* (Wakker) Boedijn	2.7 ± 0.11	5.1 ± 0.35	0.0000014	1.3 ± 0.16	2.1 ± 0.25	0.0000269
*Fusarium moniliforme* Sheldon	0.5 ± 0.21	1.9 ± 0.22	0.000073	–	1.1 ± 0.26	0.00000116
*F.solani* (Mart.) Sacc	1.2 ± 0.15	3.1 ± 0.12	0.0000021	0.3 ± 0.16	0.7 ± 0.13	0.000043
*Helminthosporium oryzae* Breda de Haan	5.2 ± 0.06	11.1 ± 0.25	0.0000023	4.7 ± 0.09	4.7 ± 0.35	1
*Microascus cirrosus* Zukal	1.3 ± 0.23	2.3 ± 0.22	0.000042	–	–	–
*Nigrospora oryzae* (Berk. & Broome) Petch	4.1 ± 0.13	9.9 ± 0.18	0.000000016	1.3 ± 0.15	6.1 ± 0.11	0.000000025
*Pyricularia grisea* Sacc	2.7 ± 0.25	5.1 ± 0.23	0.00000075	1.3 ± 0.24	2.1 ± 0.12	0.0000845

–: Absence of fungal species; US: Unsterilized seeds; SS: Sterilized seeds.

*Values given are mean of three replicates; SD = Standard Deviation.

Significant values are in bold.

**Table 6:** Intensity of insect in stored food seeds (wheat, rice) from places of Eastern UP viz., Basti, Deoria, Gorakhpur, Maharajganj, Siddhartha Nagar and Gurgaon district Haryana viz., Farrukhnagar, Manesar, Pataudi, Sohna, Bilaspur.

Name of places from which food grains samples were collected	Presence/absence of insects in stored food grains and pulses	
	Wheat	Rice
Basti	++	++
Deoria	+	+
Gorakhpur	−	+
Maharajganj	+	+
Siddhartha Nagar	−	+
Farrukhnagar	+	+
Manesar	+	+
Pataudi	+	+
Sohna	−	−
Bilaspur	−	−

− Nil.

Time to time previous investigators have reported observations on presence of storage insects on common seeds/grains. *Tribolium castaneum* (Herbst) (Coleoptera: Tenebrionidae) is one of the major insect pests of stored seeds^51,52^. *Tribolium castaneum* (Herbst) (Coleoptera: Tenebrionidae), is the most important stored-product insect pest infesting rice (*Oryza sativa* L.)^53^.

As evident from Table 7, *Aspergillus flavus*, *A. niger, Tribolium castaneum* L*.* played important role in wheat seed weight loss and seed germination. The *Aspergillus flavus* inoculated wheat seeds showed 42.15 ± 0.19, *A. niger* 39.12 ± 0.14, *Tribolium castaneum* L 37.13 ± 0.16% Carbohydrate. content respectively. The *Aspergillus flavus* inoculated seeds showed 6.7 ± 0.14, *A. niger* 5.9 ± 0.15 while *Tribolium castaneum* L*.* inoculated wheat seeds showed 4.7 ± 0.19% protein content respectively (Table 7). Results reported that *A. niger* filtrate has a adverse effect on the germination rate of wheat seeds and the development of their seedlings. It could be due to the ability of the fungus to produce Aflatoxins. These findings are consistent with previous findings. Ijaz et al.^54^ reported *A. niger* as the most damageable storage fungi among fungal pathogens which leads to lower quality and seed germination. Culture filtrates of *Aspergillus* sp. have reported in causing a reduction in seed germination and root-shoot elongation^55^. The germination rate of wheat grains irrigated with the filtrate of *A. niger* and *Rhizopus* sp. was 20% and 80% respectively, compared with 100% of the control grains, which were irrigated with water. The culture filtrates of both *A. niger* and *Rhizopus* sp. affect not only percentage of grains germination but also the morphology of wheat seedlings^56^.

**Table 7:** Fungal species/insect species vis-à-vis weight loss, germination and Nutritional composition of mature wheat seeds.

Fungal species/insect	Weight loss (in/g)		Germination%		Carbohydrates%		Protein%	
	Control	Treatment	Control	Treatment	Control	Treatment	Control	Treatment
*Aspergillus flavus*	Nil	0.179 ± 0.21	90.43 ± 0.11	50.57 ± 0.12	70.78 ± 0.10	42.15 ± 0.19	12.0 ± 0.10	6.7 ± 0.14
*A. niger*	–	0.157 ± 0.12	89.43 ± 0.11	47.30 ± 0.15	–	39.12 ± 0.14	–	5.9 ± 0.15
*Tribolium castaneum* L.	–	0.78 ± 0.14	92.00 ± 0.18	49.24 ± 0.126	–	37.13 ± 0.16	–	4.7 ± 0.19

– Nil; *Values given are mean of five replicates; SD = Standard Deviation.

**Figure 1:**
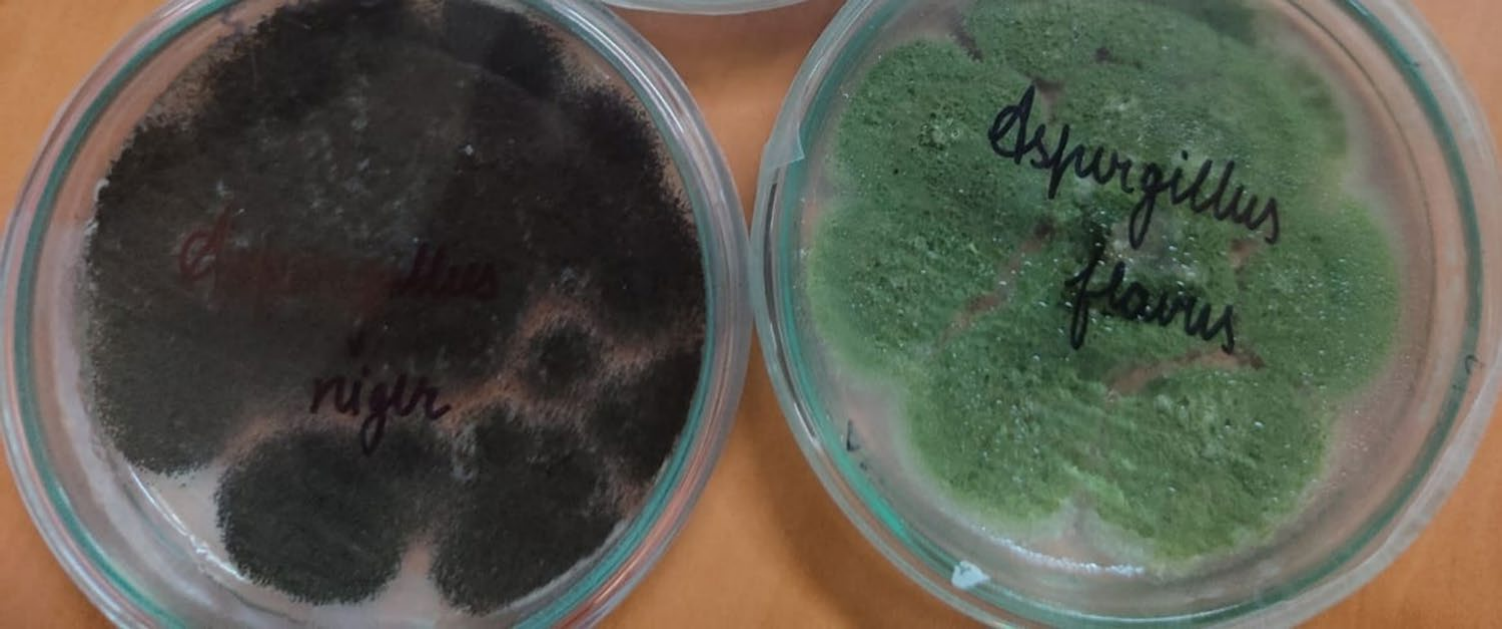
A look on dominant fungi appearing on seeds of wheat and rice.

As evident from Table 8, *Aspergillus flavus*, *A. niger, Sitophilus oryzae* L. played important role in seed rice weight loss and seed germination. The *Aspergillus flavus* inoculated rice seeds showed 41.17 ± 0.11, *A. niger* 37.11 ± 0.12, *Sitophilus oryzae* 33.13 ± 0.13% Carbohydrate. content respectively. The *Aspergillus flavus* inoculated rice seeds showed 4.7 ± 0.11, *A. niger* 4.1 ± 0.10 while *Sitophilus oryzae* L*.* inoculated rice seeds showed 4.9 ± 0.10% protein content respectively (Table 8). It is evident from investigations that *Aspergillus flavus*, *A. niger* were dominant fungi causing harm to seeds of wheat and rice (Fig. 1). A study reported that there was high negative significant correlation between seed infestation by microflora and seed germination^57^. Jalander and Gachande^55^ by study on the effect of different fungal species of seed-borne fungi of *Aspergillus* on germination and seedling growth of Bean and Cereals reported that *A. niger* caused reduction in germination percentage, growth of the plumule and radicle. As a negative impact, *A. niger* fungi affects on all rice seed germination characteristic more than all other fungi. In accordance with the results of the present study, Islam et al*.*^58^ stated that there is negative and significant correlation [R = −97%] between rate of fungal contaminations and germination percentage in different rice cultivars. Among the studied factors, *A. niger* had high negative impact compared to other factors on all rice seed germination characteristics^59^.

**Table 8:** Fungal species/insect species vis-à-vis weight loss, germination and Nutritional composition of mature rice seeds.

Fungal species/insect	Weight loss (in/g)		Germination%		Carbohydrates%		Protein%	
	Control	Treatment	Control	Treatment	Control	Treatment	Control	Treatment
*Aspergillus flavus*	Nil	0.189 ± 0.21	86.43 ± 0.11	44.57 ± 0.13	79.78 ± 0.10	41.17 ± 0.11	8.0 ± 0.10	4.7 ± 0.11
*A. niger*	–	0.177 ± 0.12	87.43 ± 0.11	49.30 ± 0.12	–	37.11 ± 0.12	–	4.1 ± 0.10
*Sitophilus oryzae* L.	–	0.06 ± 0.14	90.00 ± 0.18	48.24 ± 0.12	–	33.13 ± 0.13	–	4.9 ± 0.10

– Nil; *Values given are mean of five replicates; SD = Standard Deviation.

As evident from Table 9, the randomly selected A. flavus isolate 1 of wheat showed higher potential of aflatoxin B_1_ production (1392.940 μg/l) while rice isolate 2 showed 1231.117 μg/l production. While for both some isolates were non toxigenic. Other A. flavus isolates isolated from wheat and rice showed lower level of aflatoxin production.

**Table 9:** Aflatoxigenic potential of *A. flavus* isolates of common food grains (wheat, rice).

Fungal isolate	Food grains/pulses	Toxigenicity	AFB_1_ Content μg/l
*A. flavus* 1	Wheat	toxigenic	1392.940 ± 0.20
*A. flavus* 2	Wheat	non toxigenic	–
*A. flavus* 3	Wheat	toxigenic	1112.230 ± 0.23
*A. flavus* 4	Wheat	non toxigenic	–
*A. flavus* 1	Rice	toxigenic	1013.239 ± 0.22
*A. flavus* 2	Rice	toxigenic	1231.117 ± 0.27
*A. flavus* 3	Rice	non toxigenic	–
*A. flavus* 4	Rice	non toxigenic	–

– Nil.

Aflatoxins (AFs) are a group of mycotoxins produced as secondary metabolites by the spoilage of Aspergillus fungi, particularly Aspergillus flavus and Aspergillus parasiticus^60^. The most important members are aflatoxin B_1_ (AFB_1_), aflatoxin B_2_ (AFB_2_), aflatoxin G_1_ (AFG_1_) and aflatoxin G_2_ (AFG_2_). They are highly toxic and carcinogenic compounds that cause disease in livestock and humans^61^. In recent years, numerous studies have revealed high levels of aflatoxins and fungal contamination in rice in many countries^62^. The maximum AFB1 concentration of 606 microg kg(−1) was observed in a wheat sample from the state of Uttar Pradesh^63^. AFB1 contamination in rice ranged from 0.014 to 0.123 µg/kg^64^. Out of 1200 rice samples, 67.8% showed AFB1 ranging from 0.1 to 308.0 microg/kg. All the paddy samples from Chattishgarh, Meghalaya and Tamil Nadu showed AFB1 contamination. Milled rice grains from different states showed below the permissible levels of AFB1 (average 0.5–3.5 micro g/kg)^65^.

## Data Availability

The datasets used and/or analysed during the current study available from the corresponding author on reasonable request.

## References

[1] Bhargava MC, Kumawat KC (2010). Pests of Stored Grains and Their Management.

[2] Arthur FH, Throne JE (2003). Efficacy of diatomaceous earth to control internal infestations of rice weevil and maize weevil (Coleoptera: Curculionidae). J. Econ. Entomol..

[3] Phillips TW, Throne JE (2009). Biorational approaches to managing stored-product insects. Annu. Rev. Entomol..

[4] Wijayaratne LKW, Arthur FH, Whyard S (2018). Methoprene and control of stored-product insects. J. Stored Prod. Res..

[5] Singh B, Kaur A (2018). Control of insect pests in crop plants and stored food grains using plant saponins: A review. LWT Food Sci. Technol..

[6] You C, Guo S, Geng Z, Zhang W, Liang J, Zhang Z, Deng Z (2017). Repellent activity of compounds from *Murraya alata* Drake against *Tribolium castaneum*. Ind. Crops Prod..

[7] Mukherjee, S. https://www.business-standard.com/article/economy-policy/india-wheat-output (2022).

[8] Pathak, H., Tripathi, R., Jambhulkar, N.N., Bisen, J.P and Panda B.B. Eco-regional rice farming for enhancing productivity, profitability and sustainability. NRRI Research Bulletin No. 22, ICAR-National Rice Research Institute, Cuttack, pp 28 (2020).

[9] Palumbo R, Crisci A, Venâncio A, Abrahantes JC, Dorne JL, Battilani P, Toscano P (2020). Occurrence and co-occurrence of mycotoxins in cereal-based feed and food. Microorganisms.

[10] Shabana YM, Rashad YM, Ghoneem KM, Arafat NS, Aseel DG, Qi A, Richard B, Fitt B (2021). Biodiversity of pathogenic and toxigenic seed-borne mycoflora of wheat in egypt and their correlations with weather variables. Biology.

[11] Kumar D, Singh K, Shamim M, Siddiqui W, Srivastava D, Kumar S, Kumar R, Upadhyay P (2020). Storage of fungi with rice (*Oryza sativa*)-PRH 10 and their influence on seed quality. Indian J. Agric. Sci..

[12] Li W, Cui J, Li J, Guo J, Huang T, Zhang J, Hu H, Liu X (2022). Analysis of the Fungi community variation during rice storage through high throughput sequencing. Processes.

[13] ISTA. International rules for seed testing. Bassersdorf, Switzerland, International Seed Testing Association (2011).

[14] ISTA. International rules for seed testing. Seed Sci. Technol. **21**, 1–288 (1993).

[15] Muskett AE (1948). Technique for the examination of seed for the presence of seed borne fungi. Trans. Br. Mycol. Soc..

[16] De T (1953). The blotter method of seed health testing. Proc. Int. Seed Test Ass..

[17] Gupta S, Banerjee AB (1970). A rapid method of screening antifungal antibiotic producing plants. Indian J. Exp. Biol..

[18] Raper KB, Fennel DI (1965). The Genus Aspergillus.

[19] Raper KB, Thom C (1949). A Manual of Penicillia.

[20] Ellis MB (1971). Dematiaceous, Hyphomycetes.

[21] Ellis MB (1976). More Dematiaceous, Hyphomycetes.

[22] Hedge, J. E., & Hofreiter, B. T. In: Carbohydrate Chemistry 17 (Eds Whistler R L and Be Miller, J N) Academic Press New York (1962).

[23] Lowry OH, Rozenbrough NJ, Farr AL, Randall RJ (1951). Protein measurement with the folin phenol reagent. J. Biol. Chem..

[24] Sinha KK, Sinha AK, Prasad G (1993). The effect of clove and cinnamon oils on growth and afl atoxin production by Aspergillus flavus. Lett. Appl. Microbiol..

[25] Dwivedy AK, Singh VK, Prakash B, Dubey NK (2018). Nanoencapsulated *Illicium verum* Hook. F. essential oil as an effective novel plant-based preservative against aflatoxin B1 production and free radical generation. Food Chem. Toxicol..

[26] Tian J, Huang B, Luo X, Zeng H, Ban X (2012). The control of *Aspergillus flavus* with *Cinnamomum jensenianum* Hand.-Mazz essential oil and its potential use as a food preservative. Food Chem..

[27] El-Kady IA, Abdel-Hafez SII, El-Maraghy SS (1982). Contribution to the fungal flora of cereal grains in Egypt. Mycopathologia.

[28] Mazen MB, Abdel-Hafez SII, Shaban GMM (1984). Survey on the mycoflora of Egyptian wheat grains and their lemmae and paleae. Mycopathologia.

[29] Rajput MA, Pathan MA, Lodhi AM, Shah GS, Khanzada KA (2005). Studies on seed-borne fungi of wheat in Sindh province and their effect on seed germination. Pak. J. Bot..

[30] Fakhrunnisa M, Hashmi H, Ghaffar A (2006). Seed borne mycoflora of wheat, sorghum and barley. Pak. J. Bot..

[31] Pathak N, Zaidi RK (2013). Studies on seed-borne fungi of wheat in seed health testing programme. Arch. Phytopathol. Plant Prot..

[32] Baka ZA (2014). Plant extract control of the fungi associated with different Egyptian wheat cultivars grains. J. Plant Prot. Res..

[33] Mehboob S, Rehman A, Ali S, Idrees M, Zaidi SH (2015). Detection of wheat seed mycoflora with special reference to *Drechslera sorokiniana*. Pak. J. Phytopathol..

[34] Adhikari P, Khatri-Chhetri G, Shrestha S, Marahatta S (2016). Study on Prevalence of mycoflora in wheat seeds. Turk. J. Agric. Food Sci. Technol..

[35] Dhakar H, Ratnoo RS, Jat A (2018). Detection and identification of seed borne mycoflora of wheat (*Triticum aestivum* L.) seed samples. J. Pharmacogn. Phytochem..

[36] Pospelov S, Pospelova A, Kovalenko N, Sherstiuk N, Zdor V (2020). Biocontrol of mycoflora of winter wheat seeds. E3S Web Conf..

[37] Hussain M, Ghazanfar MU, Hamid MI, Raza M (2013). Seed borne mycoflora of some commercial wheat (*Triticum aestivum*) cultivars in Punjab, Pakistan. ESci J. Plant Pathol..

[38] Duraiswamy VS, Mariappan V (1983). Rice grain discoloration. Intern. Rice Res. Newslett..

[39] Khan SAJ, Khanzada AK, Sultana N, Aslam M (1988). Evaluation of seed health testing techniques for the assessment of seed borne mycoflora of rice. Pak. J. Agric. Res..

[40] Jeyanandarajah P, Seneviratne SN (1991). Fungi seed borne in rice in Sri Lanka. Seed Sci. Technol..

[41] Desjardins AE, Manandhar HK, Plattner RD, Manandhar GG, Poling SM, Maragos CM (2000). *Fusarium* species from Nepalese rice and production of mycotoxins and gibberellic acid by selected species. Appl. Environ. Microbiol..

[42] Nguefack J, Nguikwie SK, Fotio D, Dongmo B, Leth V, Nkengfack AE, Amvam ZPH (2007). Fungicidal potential of essential oils and fractions from *Cymbopogon citratus*, *Ocimum gratissimum* and *Thymus vulgaris* to control *Alternaria padwickii* and *Bipolaris oryzae*, two seed-borne fungi of rice (*Oryza sativa* L.). J. Essent. Oil Restit..

[43] Haque AHMM, Akhon M, Islam MA, Khalequzzaman KM, Ali MA (2007). Study on Seed Health, germination and seedling vigor of farmers produced rice seeds. Intl. J. Sustain. Crop Prod..

[44] Gopalakrishnan C, Kamalakannan A, Valluvaparidasan V (2009). Survey of seed-borne fungi associated with rice seeds in Tamil Nadu, India. Libyan Agric. Res. Cent. J. Int..

[45] Habib A, Javed N, Sahi ST, Waheed M (2012). Detection of seed borne mycoflora of different coarse and fine rice varieties and their management through seed treatments. Pak. J. Phytopathol..

[46] Ashfaq M, Shaukat MS, Akhter M, Haider MS, Mubashar U, Hussain SB (2015). Comparison of fungal diversity of local and exotic rice (*Oryza sativa* L.) Germplasm for their seed health. J. Anim. Plant.

[47] Signaboubo S, Noumbo G, Jules-Roger K (2016). Seed-borne fungi associated with rice seeds varieties in Bongor, Chad Republic. Int. J. Curr. Microbiol. Appl. Sci..

[48] Naher L, Ali MA, Sheheli S (2016). Effect of seed treatment on seed borne fungi of rice. Progress. Agric..

[49] Kumar NR, Kumar MA, Mohanapriya R (2017). Survey of seed borne fungi associated with seeds of rice in Tamilnadu. Oryza Int. J. Rice.

[50] Bertuzzi T, Romani M, Rastelli S, Giorni P (2019). Mycotoxins and related fungi in Italian paddy rice during the growing season and storage. Toxins.

[51] Arthur FH, Bean SR, Smolensky D, Gerken AR, Siliveru K, Scully ED, Baker N (2020). Development of *Tribolium castaneum* (Herbst) (Coleoptera: Tenebrionidae) on sorghum milling fractions. J. Stored Prod. Res..

[52] Gerken AR, Campbell JF (2020). Oviposition and Development of *Tribolium Castaneum* Herbst (Coleoptera: Tenebrionidae) on different types of flour. Agronomy.

[53] Buckman KA, Campbell JF, Subramanyam B (2013). Tribolium castaneum (Coleoptera: Tenebrionidae) associated with rice mills: fumigation efficacy and population rebound. J. Econ. Entomol..

[54] Ijaz A, Anwar SA, Riaz A, Khan MSA (2001). Seed-borne pathogens associated with wheat and their role in poor germination. Pak. J. Phytopathol..

[55] Jalander V, Gachande B (2012). Effect of fungal metabolites of some rhizosphere soil fungi on seed germination and seedling growth of some pulses and cereals. J. Sci. Res. Rep..

[56] Eltariki F, Abdulmajeed M, Seok M, Mohammed A (2019). Effect of fungal filtrates on germination of wheat grains and the biological control of these fungi using black pepper extract. Asia Pac. J. Mol. Biol. Biotechnol..

[57] Imolehin EO (1983). Rice seed borne fungi and their effect on seed germination. Plant Dis..

[58] Islam MS, Rahman H, Pervez Z, Mahmud MR, Alam A (2012). Studies on seedborne fungi in rice cultivars grown in non saline Tidal zone of Patuakhali and their effect on seed germination. Bangladesh Res. Publ..

[59] Monajjem S, Zainali E, Ghaderi-Far F, Soltani E, Chaleshtari MH (2014). Evaluation seed-born fungi of rice [*Oryza sativa* L.] and that effect on seed quality. J. Plant Pathol. Microb..

[60] Davis PL, Smoot JL (1972). Germination of *Penicillium digitatum* spores as affected by solutions of volatile components of Citrus fruits. Phytopath..

[61] Richard JL (2007). Some major mycotoxins and their mycotoxicoses – an overview. Int. J. Food Microbiol..

[62] Ok HE, Kim DM, Kim D, Chung SH, Chung M, Park KH, Chun HS (2014). Mycobiota and natural occurrence of aflatoxin, deoxynivalenol, nivalenol and zearalenone in rice freshly harvested in South Korea. Food Control.

[63] Toteja G, Mukherjee A, Diwakar S, Singh P (2006). Aflatoxin B 1 contamination in wheat grain samples collected from different geographical regions of India: A Multicenter Study. J. Food Protect..

[64] Najeeb S, Al-Zoreky F, Saleh A (2019). Limited survey on aflatoxin contamination in rice. Saudi J. Biol. Sci..

[65] Reddy KR, Reddy CS, Muralidharan K (2009). Detection of *Aspergillus* spp. and aflatoxin B1 in rice in India. Food Microbiol..

